# Inconsistent use of gesture space during abstract pointing impairs language comprehension

**DOI:** 10.3389/fpsyg.2015.00080

**Published:** 2015-02-09

**Authors:** Thomas C. Gunter, J. E. Douglas Weinbrenner, Henning Holle

**Affiliations:** ^1^Department of Neuropsychology, Max Planck Institute for Human Cognitive and Brain SciencesLeipzig, Germany; ^2^Department of Psychology, University of HullHull, UK

**Keywords:** pointing, gesture, N400, P600, communication, referent identification

## Abstract

Pointing toward concrete objects is a well-known and efficient communicative strategy. Much less is known about the communicative effectiveness of abstract pointing where the pointing gestures are directed to “empty space.” McNeill's (2003) observations suggest that abstract pointing can be used to establish referents in gesture space, without the referents being physically present. Recently, however, it has been shown that abstract pointing typically provides redundant information to the uttered speech thereby suggesting a very limited communicative value (So et al., 2009). In a first approach to tackle this issue we were interested to know whether perceivers are sensitive at all to this gesture cue or whether it is completely discarded as irrelevant add-on information. Sensitivity to for instance a gesture-speech mismatch would suggest a potential communicative function of abstract pointing. Therefore, we devised a mismatch paradigm in which participants watched a video where a female was interviewed on various topics. During her responses, she established two concepts in space using abstract pointing (e.g., pointing to the left when saying *Donald*, and pointing to the right when saying *Mickey)*. In the last response to each topic, the pointing gesture accompanying a target word (e.g., Donald) was either consistent or inconsistent with the previously established location. Event related brain potentials showed an increased N400 and P600 when gesture and speech referred to different referents, indicating that inconsistent use of gesture space impairs language comprehension. Abstract pointing was found to influence comprehension even though gesture was not crucial to understanding the sentences or conducting the experimental task. These data suggest that a referent was retrieved via abstract pointing and that abstract pointing can potentially be used for referent indication in a discourse. We conclude that abstract pointing has a potential communicative function.

## Introduction

One of the most fundamental and universal ways to communicate is to point in order to attract the attention of your interlocutor to a certain object or event. This seemingly simple gesture can be an effective communicative device (Enfield et al., 2007). Pointing is one of the earliest communication tools in pre-linguistic infancy and also serves as a joint attention cue thereby facilitating infant language learning (Butterworth, 2003; Tomasello et al., 2007; Liszkowski and Tomasello, 2011). Similarly, it can serve as a disambiguation cue for ambiguous utterances. Imagine that during a restaurant visit you ask the waiter for the restrooms and he replies, “They're over there.” Only when accompanied by a concrete pointing gesture would this verbal response be helpful (cf. Clark, 1992, 2003). Pointing can also change the meaning of an utterance. Kelly et al. (1999), for instance, showed that an expression like “It is hot in here” accompanied by a pointing gesture is interpreted as an indirect request to open the window. Thus, in this example the pointing changed a factual statement into a request. Compared to other gestures, pointing seems to be the most flexible gesture type because its meaning is almost entirely determined by context. Whereas emblems (like the thumbs-up gesture) are completely context independent and have clear regional meaning (Morris, 1979), the meaning of iconic gestures (like making a round shape with two hands to indicate, for instance, a ball) is somewhat context dependent although there is still some information in the form of decontextualized iconic gestures (Hadar and Pinchas-Zamir, 2004; Molnar-Szakacs et al., 2007). The flexibility of the pointing gesture allows a speaker to not only refer to concrete objects. In addition, pointing can be used to establish abstract concepts in gesture space. In the following, we will investigate this more advanced^1^ use of pointing and explore the potential communicative significance of so-called abstract pointing.

In contrast to the concrete pointing gestures where the pointing is directed to a physically present target (see the above example of the waiter), abstract pointing gestures are directed to “empty space.” Abstract pointing gestures are formally defined by their orientation toward “empty space.” The parts of space indicated by such gestures are hypothesized to temporally attain a representational value for the purpose of discourse that can be used by their perceivers to track concrete and abstract components of the discourse (McNeill, 1992). As an example, imagine a conversation about cartoon characters with a friend and she says, “As a child I used to read comic books about Donald Duck and Mickey Mouse.” While talking, she accompanies the word “Donald” with a pointing gesture to the left and the word “Mickey” with a pointing gesture to the right, although none of the characters are present. A bit later she replies, “Well, I liked these books the most,” accompanying the word “these” with a pointing gesture to the left thereby referring back to Donald Duck. In this example pointing was used in the abstract sense (For an example of natural abstract pointing use please see the Supplementary Information). Thus, during abstract pointing people employ gesture space to refer to particular discourse information, even though nothing is actually present at the indicated position. Potentially, this use of pointing could play a role in discourse build-up.

One very important component of discourse build-up is specifying the characters of a narrative in such a way that a listener can identify who is doing what to whom (Garrod, 2001). Linguistically, speakers can use, for instance, nouns and pronouns for this purpose. Another way of identifying characters in a discourse is the use of gesture space (for a discussion see Clark and Bangerter, 2004). At first glance it seems that such a strategy would work particularly well when referents are left underspecified as would be the case for the pronoun “he” and “him” in the story about Mickey Mouse and Donald Duck (“… and then he saw him on the street”). Although in rare cases speakers indeed use this strategy, So et al. (2009) suggest that abstract pointing is typically used in a different way. They found that speakers indeed frequently used gesture location to identify referents during their narratives (using iconic gestures or abstract pointing), but particularly so when these referents were also uniquely specified in their speech. In their experiment, native English speaker, all naïve to sign language, were asked to describe video materials involving protagonists of different (Man-Woman) or same gender (Man-Man). Although the speakers used the spatial location of their gestures systematically to identify their referents, they did not use gestures to compensate for the under-specification in speech in the man-man stories. Speakers identified referents in gesture reliably less often when telling the man-man (27%) story than when telling the man-woman story (62%). Additionally gestures were rarely used to compensate for the absence of lexical specificity in pronouns or nouns. Pronouns that did not uniquely specify a particular referent were only in rare cases accompanied by a gesture that identified the reference whereas pronouns that did specify a particular referent were accompanied by gestures much more often (13 vs. 55%). Thus, the data of So et al. (2009) seem to suggest that in most cases abstract pointing provides only redundant information which does not have an important communicative function and is possibly only functionally relevant for the speaker. One could, for instance, hypothesize that speakers use it for reducing their working memory load (cf. Marstaller and Burianová, 2013) or cognitive load in general (for a recent review on these issues see Pouw et al., 2014). Since abstract pointing seems to be a less informative communicative cue for the listener, we were interested in whether perceivers are sensitive at all to this gesture cue. We therefore devised a mismatch paradigm in which pointing gestures were used to establish two concepts in space, followed by a critical sentence in which the pointing gesture either matched or mismatched the previously established location. Note that our paradigm resembles the situation as described by So et al. (2009). Except for the mismatch situation, abstract pointing was also redundant with the accompanying speech since they both relate to the same referents. If abstract pointing is mainly beneficial for the speakers and has no communicative value, a mismatch should not have any impact on the listener. Alternatively, if there is an obligatory interaction between gesture and speech during comprehension, as argued by Kelly et al. (2010a), a mismatching abstract pointing gesture should impair language comprehension. Thus, although our paradigm does not directly test the impact of gestures on comprehension processes *per se*, it will establish whether a mismatch between gesture and speech is detected or not. If it is, this finding opens the possibility that abstract pointing plays a role in language comprehension.

In the present experiment, the brain's response to gesture-speech stimuli was measured using event related potentials (ERPs) taken from the electroencephalogram. Since we explored the potential impact of gesture on language processing, two language-associated ERP components, namely the N400 and the P600, are important within the scope of this paper. The N400 is a negativity which peaks roughly 400 ms after the onset of a potentially meaningful stimulus such as words or pictures. It has been suggested that the N400 reflects the ease of retrieving information about an encountered stimulus (e.g., lexical and semantic knowledge) from long-term memory. The easier this retrieval, the smaller the N400 (for reviews see Kutas and Federmeier, 2000; Lau et al., 2008; Kutas and Federmeier, 2011). Retrieval is, for instance, facilitated when a word is pre-activated by a prior context leading to a reduced N400. Note that N400 effects have been observed in virtually all ERP studies on gesture processing, both in violation (Kelly et al., 2004, 2007, 2010b, 2012; Wu and Coulson, 2005, 2007, 2010, 2011; Özyürek et al., 2007; Sheehan et al., 2007; Cornejo et al., 2009; Ibanez et al., 2010, 2011; Habets et al., 2011) as well as in disambiguation paradigms (Holle and Gunter, 2007; Obermeier et al., 2011, 2012). Only one study investigating the impact of beat gestures^2^ on syntactic processing reported an effect of the P600 component (Holle et al., 2012)^3^.

The P600 is a positive deflection which peaks roughly 600 ms after the onset of a critical stimulus, and is traditionally thought to be related to syntax processing costs (e.g., Osterhout and Holcomb, 1992; Hagoort et al., 1993). Recent studies have shown, however, that P600-like effects can also be elicited by semantic manipulations (e.g., Kuperberg et al., 2003; Hoeks et al., 2004; Kim and Osterhout, 2005). One way to explain the semantic P600 is to assume that it reflects a reanalysis of the stimulus material, which is triggered by a conflict between two different processing streams (for reviews see Kuperberg, 2007; van de Meerendonk et al., 2009). In line with this, van Herten et al. (2006), for example, propose that speech is analyzed by a standard syntactic process and by a heuristic process. Although these multi-stream models are a very useful approach, a recent review by Brouwer et al. (2012) suggests that none of these models account for all relevant data and return to a single-stream architecture. They propose a more general hypothesis and suggest that the P600 reflects the reorganization or the updating of a Mental Representation of what is being Communicated, the so-called MRC. An MRC is a representation of how a person interprets the current communicative situation. Brouwer et al. (2012) suggest that this mental representation is constantly refined based on the incoming input. The amplitude of the P600 is supposed to reflect the difficulty of this process. If a new word requires a more considerable modification of the mental representation, this will result in an increased P600.

In order to explore whether perceivers are actually sensitive to abstract pointing gestures, we set up a mismatch paradigm in which participants were presented with video material showing an interview situation where, within each discussed topic, abstract pointing established the representation of two referents in gesture space (i.e., Donald Duck on the left, Mickey Mouse on the right). At the end of each topic, participants were presented with an experimental sentence which was accompanied by an abstract pointing gesture that was either congruent (pointing to the left while saying “Donald”) or incongruent (pointing right while saying “Donald”) to the previously established location. If abstract pointing has a potential communicative value, processing the target should be more difficult when gesture and speech provide mismatching information. If speech refers to Donald but the location of the abstract pointing gesture indicates that Mickey is the referent, one has to assume that if abstract pointing is taken into account by the recipient, more effortful memory retrieval is needed since two referents are being retrieved. We therefore hypothesized that a more negative N400 would be elicited in the violation condition when abstract pointing is taken into account. Additionally, if pointing gestures are potentially communicative, being presented with conflicting linguistic and gestural information might trigger additional reanalysis and reorganization costs, as indicated by a P600 effect.

## Methods

### Participants

Thirty-four German native-speaking students were paid for their participation. They gave written informed consent following the guidelines of the Ethics committee of the University of Leipzig, in accordance with the declaration of Helsinki. Two participants were excluded from further analyses because of excessive artifacts. The remaining 32 participants (half female; mean age 24.6, age range 19–30 years) were right-handed, had a mean laterality quotient of 94.4 (*SD* = 7.2) (Oldfield, 1971), and reported neither a hearing impairment nor a history of neurological impairment.

### Experimental design

Each participant had to watch an ongoing interview between an interviewer (one of the experimenters: J. E. D. W.) and an interviewee (a professional actress). During the interview 84 topics were presented. All topics were of a dualistic nature, meaning that there were always two opposing options to talk about, with the interviewer prompting the interviewee to consider advantages and disadvantages of each option. Typical examples are “cats vs. dogs” or “notebook vs. desktop computer.”

Each topic consisted of two phases, an establishing phase and a critical phase. The goal of the former was to establish a gesturing order. During the topic “Donald vs. Mickey,” for instance, the interviewee conducted several abstract pointing gestures with the left hand to the left side while talking about Donald and with the right hand to the right side while talking about Mickey. Thus, the pointing gestures established the concept *Donald* in the left and the concept *Mickey* in the right gesture space of the speaker. During the critical phase, a last question was asked and the interviewee responded to it with a verbal utterance that was accompanied by a left or right abstract pointing gesture. In one condition the referent indicated by speech and gesture location were congruent, in the other condition they were incongruent. Thus, in the congruent condition gesture and speech refer to the same lexical entry whereas for incongruent trials they refer to two different entries. Each topic existed in four possible versions (congruent/incongruent abstract pointing, left/right hand pointing); an example is given in Table 1. In contrast to the response of the critical phase, the establishing phase and the question of the critical phase were always identical between the versions of a certain topic. Every participant watched a specific topic only in one version. Between participants the versions of a specific topic were counter-balanced.

**Table 1:** **Example for all versions of a topic's critical response in experiment 1**.

**Gesture congruency**	**Pointing side**	**Response**
Congruent	Left	Soweit ich weiß, ist *Donald*_[Donald]_ später erfunden worden^a^
		As far as I know, *Donald*_[Donald]_ was created later^b^
Congruent	Right	Soweit ich weiß, ist *Micky*_[Micky]_ zuerst erfunden worden^a^
		As far as I know, *Mickey*_[Mickey]_ was created earlier^b^
Incongruent	Left	Soweit ich weiß, ist *Micky*_[Donald]_ später erfunden worden^a^
		As far as I know, *Mickey*_[Donald]_ was created earlier^b^
Incongruent	Right	Soweit ich weiß, ist *Donald*_[Micky]_ später erfunden worden^a^
		As far as I know, *Donald*_[Mickey]_ was created later^b^

ERPs were measured at the onset of the italicized words. Subscriptions contain the referent indicated by abstract pointing.

a Original.

b Translation.

In order to avoid predictability of the critical phase, one third of the topics had an establishing phase of two gestures, another third had three gestures, and the last third had four gestures per side. Within a topic each side had the same number of establishing gestures. In contrast to the variables congruency and side, each topic existed only in one version regarding the number of establishments. Referring to the first three variables (congruency, side, number of establishments), the topics were presented in a pseudo randomized order. The randomization of the factor side ensured that across participants there was no consistent left or right pointing bias which could be coupled to the preference of the actress (cf. Casasanto and Jasmin, 2010). The experiment was carried out in two sessions. Half of the participants watched topics 1–42 during the first session and topics 43–84 during the second session; for the other participants the order was reversed.

### Interview preparation

In a first step, 90 topics of dualistic nature were selected. They were sent to the actress in order to familiarize her with them. Subsequently, we determined the questions the interviewer was supposed to ask during the establishing phase. We also looked for arguments the interviewee could potentially use for her responses. Please note that we did not prepare fully scripted answers for the establishing phase. Instead we wanted to be able to give the actress information at hand in cases where she did not have the knowledge to answer a question.

Additionally, the question-answer pair for the critical phase was prepared. There were two spoken versions of the response, one for each option (see Table 1). In contrast to the establishing phase, we prepared fully scripted responses for both response options, because it was our goal to have the same wording until the critical word when the abstract pointing took place.

### Equipment, shooting, and post-production of the stimulus material

**Figure 1:**
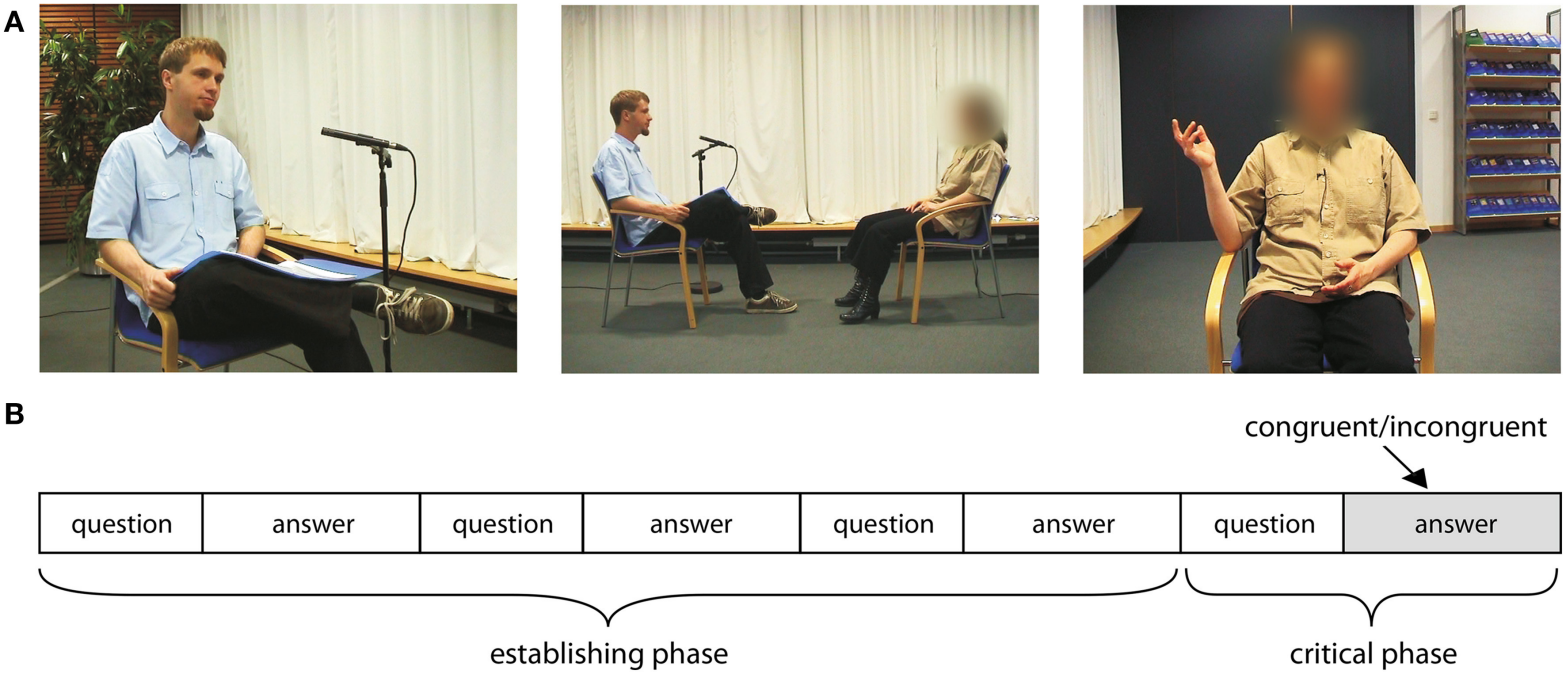
**(A)** Shows the three different shots used for videotaping. The left picture shows the medium shot of the interviewer, the middle picture the long shot, and the right picture the medium shot of the interviewee conducting an abstract pointing gesture. **(B)** Shows a graphical representation of the basic structure of an interview topic.

A “Sony DCR-TV60E” consumer camera was used for the recordings of the video stimuli. The recording format was DV-PAL, the videos had a resolution of 720 × 576, they were progressive, had a frame rate of 25 frames per second, and the aspect ratio was 4:3. The audio signal was recorded separately with a “Roland CD-2 CF/CD Recorder” and was saved as wav-files at 44.1 kHz. A clapperboard was used for the synchronization of the video and audio materials during post-production. Three different shots were used for videotaping (see Figure 1). In general, the topics were filmed in no specific sequence and no references were made between topics. Sometimes, however, an additional version of a question was recorded, where references to other parts took place. When applied in the final material, these questions increased the sense of interaction between the interview partners. Final Cut Pro 5.1.4 was used for video post-production. For the incongruent versions, we simply switched the audio tracks of the congruent versions. Because this procedure destroyed the lip synchrony, we blurred the face of the interviewee (cf. Figure 1). Since Levelt et al. (1985) showed that the gap between the apex of a pointing gesture and the target word onset is rather small (i.e., 53 ms), we decided to synchronize these time points of both the congruent and the incongruent conditions by aligning the audio track to the video track. The achieved precision lies within one video frame. During the experiment, the participants were told that the blurring was needed to keep the interviewee anonymous. In order to get a coherent interview, topics with a similar subject were grouped into “meta-topics.” For instance, the topics “PC vs. Mac” and “Linux vs. Windows” were combined into the meta-topic “computers.” Eventually, the 84 topics were distributed over 20 meta-topics, each containing two to seven single topics. All videos were compressed in the Audio Video Interleave (AVI) format. Xvid was used as the video codec and MP3 as the audio codec. A total of approximately 35 h of raw video footage were used to create the stimulus material.

### Procedure

In order to keep the participants attentive throughout the experiment, a memory task was included. After each meta-topic, the participants had to answer three dual-choice questions about the preceding video content. Since there were 20 meta-topics, the participants had to answer a total of 60 memory questions by pressing a key, after which immediate feedback was given. Participants were instructed to put the emphasis on accuracy, not speed. The memory task was neither about the content of a topic's critical phase nor was it gesture related. For instance for the topic “computer vs. laptop,” participants were asked: “Which pointing device is used by Sabine?” Responses were given via a button press. In this case the right button press meant “Touchpad”; left button press meant: “TrackPoint.” The participants were sitting in a dimly-lit room and were informed about the EEG's susceptibility to artifacts from body and eye movements. Following each video, there was a pause of self-determined length. The videos subtended approximately 9° visual angle horizontally and 7° vertically. Each experimental session lasted approximately 2.5 h.

### EEG recording and analysis

The EEG was recorded using 59 Ag/AgCl electrodes which were located according to sites defined in the extended 10-20 system of the American Clinical Neurophysiology Society (2006). Sternum served as ground. The EEG was amplified using a PORTI-32/MREFA amplifier (DC to 135 Hz) and digitized on-line at 500 Hz. Impedances were kept below 5 kΩ. During data acquisition, the EEG was referenced against the left mastoid electrode; a linked mastoid reference was calculated off-line. The electrooculogram (EOG) was measured horizontally as well as vertically.

The EEG was both automatically and manually checked for artifacts. Automatic artifact rejection used a sliding time window of 200 ms. Epochs were rejected in the case of a 40 μV deviation on the EOG channels or a 50 μV deviation on the EEG channels. The mean rejection rate was 23.4% (*SD* = 12.8). On average, 31.5 (*SD* = 5.7) congruent and 32.8 (*SD* = 5.5) incongruent trials were entered into the analysis.

In the ERP analyses, single subject averages were calculated for congruent and incongruent trails. The epochs lasted from 200 ms prior to the word onset of the critical word to 1000 ms afterwards. A 200 ms pre-stimulus baseline was applied. Ten Regions of Interest (ROIs) were defined: anterior outer left: AF7, F5, FC5; anterior inner left: AF3, F3, FC3; anterior central: AFZ, FZ, FCZ; anterior inner right: AF4, F4, FC4; anterior outer right: AF8, F6, FC6; posterior outer left: CP5, P5, PO7; posterior inner left: CP3, P3, PO3; posterior central: CPZ, PZ, POZ; posterior inner right: CP4, P4, PO4; posterior outer right: CP6, P6, PO8. The N400 was analyzed using a time window between 200 and 450 ms whereas the time window for the P600 was between 600 and 800 ms.

A repeated measures ANOVA using Session (first, second), Congruency (congruent, incongruent), ROI (outer left, inner left, center, inner right, outer right), and Ant/Pos (anterior, posterior) as within-subject variables was calculated for each time window. Only effects which involve the crucial variable congruency will be reported. Where appropriate, corrected *p*-values were calculated (Greenhouse and Geisser, 1959).

## Results

### Memory task

In the dual choice memory task, the participants selected the correct response in 95.1% of the cases (*SD* = 4.4; ranging from 81.7 to 100.0%).

### ERPs

**Figure 2:**
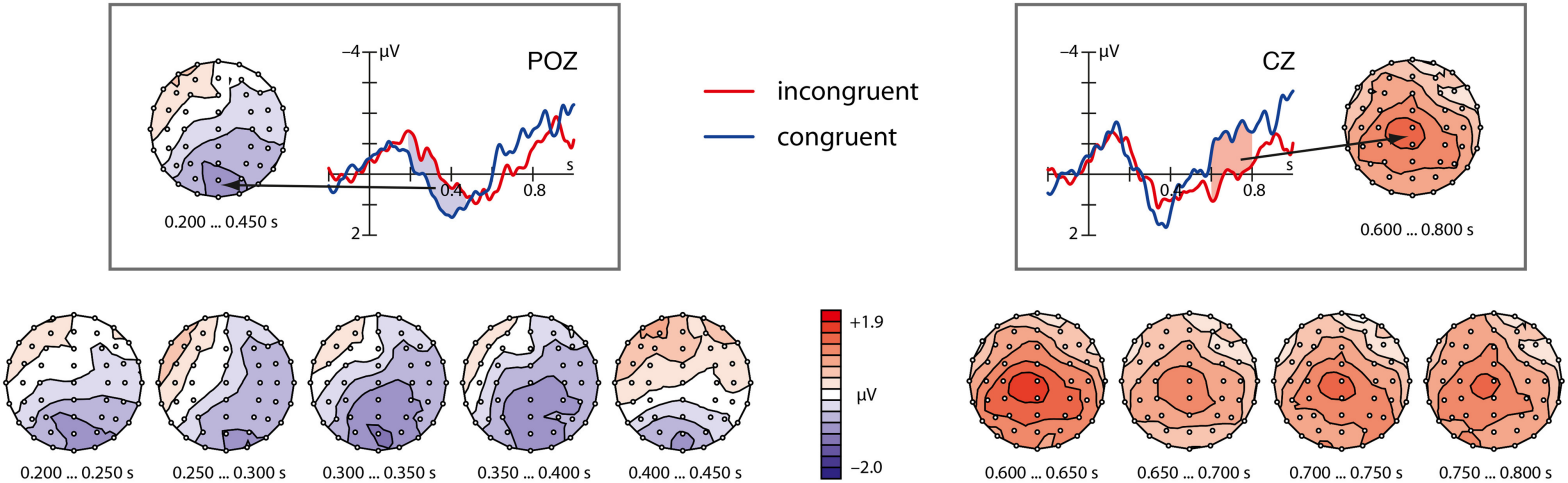
**Grand average ERPs for the matching congruent (blue) and the mismatching incongruent (red) abstract pointing gestures at the Poz and Cz electrode and the scalp distribution of their difference**. The left side depicts the N400 analysis window (blue shaded color) and the scalp distribution of the complete time window (200–450 ms) in one step and 5 consecutive 50 ms steps. The right side shows the homolog for the P600 analysis window (600–800 ms) colored in shaded red.

As can be seen in Figure 2, the incongruent condition showed both a parietally distributed N400 followed by a centrally distributed P600.

#### N400 window (200–450 ms)

The repeated measurement ANOVA with the factors Session (2), Congruency (2), Ant/pos (2), and ROI (5) revealed no significant main effect, but two significant interactions: One is a two-way interaction of Congruency × Ant/Pos with *F*_(1, 31)_ = 5.56, *p* = 0.025, the other one is a three-way interaction of Congruency × Ant/Pos × ROI with *F*_(4, 124)_ = 3.73, *p* = 0.028, ε = 0.526. A step-down analysis of the three-way interaction holding the factor Ant/Pos constant revealed at posterior sites a significant main effect of Congruency with *F*_(1, 31)_ = 4.41, *p* = 0.044. No interaction between Congruency and Session turned out to be significant. To conclude, watching a mismatching pointing gesture leads to an N400 at posterior sites. This effect did not interact with the factor Session and is thus stable across the two experimental sessions.

#### P600 window (600–800 ms)

The repeated measurement ANOVA revealed a significant main effect for Congruency [*F*_(1, 31)_ = 7.89, *p* = 0.009]. No interactions were significant. This analysis shows that mismatching abstract pointing leads to a broadly distributed P600 component independent of the experimental session.

## Discussion

In this experiment we were interested in whether abstract pointing has a potential communicative function, in particular whether it could represent referent information in a discourse as hypothesized by McNeill (1992). To our knowledge, there has only been one single case study of McNeill (2003) that supports referent indication via abstract pointing for both interlocutors of a conversation. In contrast to this observation study, the experimental production data of So et al. (2009) suggested that abstract pointing does not have a clear communicative value because it is typically redundant information being present both in speech and gesture. The present experiment showed, however, a clear difference in the recipients' brain response when a verbal utterance was accompanied by a congruent abstract pointing compared to an incongruent abstract pointing. This suggests that participants take this very advanced type of pointing into account and build associations between a certain location in gesture space and a verbal referent.

It has previously been proposed that there is a strong and obligatory interaction between iconic gestures and speech during comprehension (Kelly et al., 2010a). The present study investigated whether such an obligatory interaction does also occur between abstract pointing gestures and speech. We observed that although the information provided by abstract pointing gestures was not task-relevant, mismatching gestures modulated two well-known language-associated ERP components, the N400 and the P600 (see below). The results are therefore compatible with the idea that perceivers tend to automatically combine the information provided by gestures with the information gained from speech into a single unified representation during language comprehension. Crucially, this integration does not appear to be limited to co-speech iconic gestures, but appears to encompass other gesture types as well, including beat gestures (Holle et al., 2012) and abstract pointing gestures (the present study).

###  

#### N400-P600 pattern

When specifying the brain responses elicited by the present experiment, we see a negativity (200–450 ms) which was followed by positivity (600–800 ms) for the mismatching condition. The negativity for the incongruent condition was identified as an N400 effect^4^, the positivity as a P600. This N400-P600 pattern fits quite well with what Hoeks and Brouwer (2014) describe as the Retrieval-Integration account of language processing which suggests that language comprehension proceeds in biphasic N400-P600 cycles. Specifying the brain basis of this account, Brouwer and Hoeks (2013) suggest that the left posterior middle temporal gyrus (pMTG) is involved in the retrieval of lexical information associated with a word leading to an N400. In a next step this information is integrated with the prior context and the “Mental Representation of what is being Communicated” (MRC) is being updated. This integration process is suggested to take place in the left inferior frontal gyrus (IFG) and generates the P600. The model does not specify the functional roles of the dorsal and ventral pathways which connect the IFG and the pMTG. Note that this account resembles dual-pathway models of language processing to a large extent (see Dick and Tremblay, 2012; Friederici, 2012). Interestingly, the IFG and pMTG (more typically however the superior temporal gyrus) also play a role in gesture processing *per se* (Josse et al., 2012). Although the Retrieval-Integration account of language processing is mainly based on language data, the present study extends it to gesture processing making this model more general. It seems that incongruent abstract pointing leads to higher retrieval and integration effort as reflected in increased N400 and P600 amplitudes.

#### N400

During the perception of a conversation, our brain continuously has to retrieve information from long-term memory and—as pointed out—retrieval effort is reflected in the amplitude of the N400 (cf. Federmeier and Kutas, 1999; Gouvea et al., 2010; Kutas and Federmeier, 2011). The larger the N400, the more retrieval effort has been made. Because a clear N400 effect was found for incongruent as compared to congruent conditions, the present data suggest that retrieval of referent information from long-term memory can also be triggered by an abstract pointing gesture which has been associated with a certain referent. By repeatedly pairing an initially meaningless hand movement that points into empty space with a linguistic unit during the establishment phase, the speaker establishes a particular concept in gesture space. Once an area of gesture space has become associated with a concept, an abstract pointing gesture *per se* is sufficient to retrieve the meaning of the concept. In the congruent condition, gesture and speech refer to the same referent leading to the retrieval of only a limited amount of information from memory. In the incongruent condition, however, gesture and speech refer to different referents. Thus, compared to the congruent condition more information needs to be retrieved from memory leading to a more negative N400. The posterior scalp distribution of the N400 can be attributed to the specifics of our stimuli since it is well known that the scalp distribution of the N400 varies with stimulus type (e.g., videos: Sitnikova et al., 2003; pictures: Ganis et al., 1996; emblems: Gunter and Bach, 2004; visually presented words: Kutas and Hillyard, 1983; auditory presented words: McCallum et al., 1984; concrete vs. abstract words: Holcomb et al., 1999). For instance, in a study exploring emblems (i.e., meaningful hand postures which have a clear-cut regional meaning like the “thumbs-up” hand posture) Gunter and Bach (2004) showed that compared to emblems, meaningless but highly similar hand postures, showed beside a frontally distributed N300 component a more posteriorly distributed N400 as is typically found in picture processing. They interpreted on the basis of similarities in scalp distribution that the semantic representations of the concepts expressed by meaningful hand postures have similar properties to those of abstract word.

#### P600

The late positive deflection for the incongruent condition was classified as a P600 effect. This finding adds to the gesture literature describing gesture-speech mismatch effects as such studies typically show an enhanced N400 for the mismatch condition which is sometimes prolonged in time (for instance, up to 1000 ms in Wu and Coulson, 2007). Although traditionally the P600 was hypothesized to reflect syntactic processing, this cannot have played a role in the current experiment. The only difference between our two conditions is that in the mismatch condition speech and gesture refer to different referents. Although the simultaneous activation of two referents might be remarkable for the participants (see below) this probably does not represent a syntactic violation. Clearly anaphoric referencing has a syntactic component in that it is related to binding different syntactic elements [i.e., nouns with pronominals such as pronouns (him/her) or reflexives (himself/herself)] together at the level of a sentence (cf. Piñango and Burkhardt, 2002). In our mismatch condition both the “gesture” and the “speech” referent are of the same syntactic class and there seems to be no reasonable manner how they can be bound in a syntactic way. As discussed in the Introduction, part of the P600 literature suggests the more general notion that the P600 reflects a reanalysis triggered by a conflict between two processing streams (e.g., Kuperberg, 2007; van de Meerendonk et al., 2009). Such a notion would suggest a conflict between the processing of gesture and speech. That is, the detection of a conflict between gesture and speech could potentially trigger a reanalysis of the stimulus material leading to a more positive P600.

Alternatively, one could interpret the P600 in the context of the MRC hypothesis formulated by Brouwer et al. (2012). As discussed above, this single stream hypothesis suggests that a person constantly interprets his current communicative situation by revising or updating a “mental representation of what is being communicated” (MRC). The more effortful this process, the larger the P600. Compared to the congruent condition, where no update or revision is needed, the incongruent condition indeed represents an effortful event. Clearly both referents must already have been integrated in the MRC, on the basis of the foregoing interview where the interlocutors talked about both referents. During the incongruent condition, gesture and speech refer to different referents, which is at odds with what is typically expected from an efficient communicative situation (cf. Gricean maxims; Grice, 1989). The incongruent situation can be seen as a conflict where it is unclear which information is “correct,” the referent information indicated by the gesture or by speech. Our experiment cannot tell us which information will be preferred by the parser. The resolution of this conflict, including updating and revising the MRC, is suggested to be reflected in the P600 effect.

#### Late positivity and gestures

At first glance, it was remarkable to see that our experiment elicited a P600 component. As has already been discussed, the vast majority of ERP studies on gestures reported an isolated N400, without a subsequent P600, suggesting that there was no need for any update or revision processes in those studies and that gesture related information only needed to be retrieved from memory. This is a bit unexpected if one assumes, as Hoeks and Brouwer (2014) do, that language comprehension proceeds in biphasic retrieval-integration (N400-P600) cycles. A possible explanation relates to the fact that all of these N400 studies used iconic gestures. Iconic gestures are distinguished by their “close formal relationship to the semantic content of speech” (McNeill, 1992, p. 12). Although the form of an iconic gesture has some meaning *per se*, this type of gesture is known to have a rather vague meaning and can only be clearly interpreted in a particular context (Krauss et al., 1991; Hadar and Pinchas-Zamir, 2004), for example, when the hands of a speaker make a roundish shaped gesture such an iconic gesture could refer to an apple, a ball, the globe, etc. where the inferred meaning depends on the context. Additionally, iconic gestures can depict semantic aspects that are not covered by speech—for instance, when performing a typing action with your fingers while saying “… and then he wrote a letter,” the gesture channel reveals that a keyboard was used (Cassell et al., 1999). In all these examples it is clear that although meaning information needs to be accessed, it does not have a fundamental impact on the MRC: there is no reason to invest additional effort in establishing/revising a representation of what the speaker wants to convey. The retrieval of “new” information, as in the keyboard example, is only consolidating the semantic network^5^ which was already active in working memory on the basis of the foregoing context. In contrast, abstract pointing does not contain any generic semantic information. Abstract pointing can only refer to meaning after the establishing phase. At this point there is a clear cut and simple rule that formulates which part of gesture space relates to which referent. Consequently, when speech and gesture do not match, there is no way of solving this mismatch semantically because both referents are already active in working memory. Thus, in the case where gesture and speech refer to different referents, the MRC needs to be adapted since such a mismatch is at odds with what is pragmatically expected from a communicative situation (cf. Gricean maxims).

#### The consistent use of gesture space

One important aspect of abstract pointing is the consistent usage of gesture space. Although McNeill (1992) suggested that gesture space is used to track the referents of a discourse, the experimental literature shows a more mixed state of affairs. Several studies, typically concerned with co-speech gestures in general, have suggested that the consistent usage of gesture space is certainly a phenomenon that occurs on a regular basis, but also that the usage of gesture space is not truly reliable (McNeill and Levy, 1993; So et al., 2005, 2009; Gullberg, 2006). Gullberg (2006), for instance, explored iconic gestures which accompany a spoken object in a second language. In an analysis which examined where in gesture space the second occurrence of an iconic gesture appeared, she found that only in 42% of the time gesture space was used consistently. In a similar vein, So et al. (2009) found that approximately 35% of the time speakers used spatial location of their gestures to systematically identify referents of their story. Possibly individual differences in the consistent use of gesture space can account for such findings. A study by Alamillo et al. (2010) found that 68% of their adult participants showed consistent usage of gesture space while the rest did not. Thus, only some speakers use gesture space consistently to establish concepts in space whereas others do not. The present study showed that inconsistent use of gesture space has an immediate detrimental effect on online measures of language comprehension. More studies with different paradigms are required to investigate whether a consistent use of gesture space can also facilitate communication. Additionally, because there is the possibility in our paradigm that the blurring of the face made the actress's pointing possibly more salient than in a natural situation, it will be interesting to see in future experiments if the same results are obtained when the speaker's face is visible.

In summary, the data suggest that recipients process abstract pointing, even when pointing is not providing task-relevant information. The observed N400-P600 pattern gave a clear indication that the incongruent condition led to more memory retrieval and the effortful update of the representation of the sentence or the MRC. The most reasonable way to explain this pattern of results is to assume that indeed a referent was retrieved via abstract pointing and that abstract pointing can be used for referent indication in a discourse. To put it differently, abstract pointing has a potential communicative function and is not only used for the benefit of the speaker.

### Conflict of interest statement

The authors declare that the research was conducted in the absence of any commercial or financial relationships that could be construed as a potential conflict of interest.

## Footnotes

^1^It is therefore not surprising that although concrete pointing is the first of all gesture types being acquired around the age of one year (Liszkowski and Tomasello, 2011), abstract pointing is thought to develop much later somewhere between the age of 8 and 11 years (McNeill, 1992), suggesting a much more complex underlying mechanism for this gesture type. Note that a similar reference-related usage of space is present in sign language, suggesting an ecological usefulness of abstract pointing gestures (Neidle et al., 2000; Emmorey and Falgier, 2004); for a recent critical discussion on pronouns and pointing in sign language see Cormier et al., 2013).

^2^Beat gestures are short, rhythmic hand movements that match the cadence of speech (Hubbard et al., 2009). They are suggested to accent or emphasize portions of their co-expressive speech (Efron, 1941/1972).

^3^Note that there have been some reports of a late positive component for gesture studies exploring the effects of gestures on metaphoric and figurative language processing (Cornejo et al., 2009; Ibanez et al., 2010, 2011). It is possible that these effects relate to the pragmatic use of language which is known to elicit a P600 (Regel et al., 2011).

^4^Note that there are some studies (cf. van Berkum et al., 1999) that have identified a specific component related to referent processing. This so-called Nref effect is broadly distributed with a distinct frontal focus starting around 200 ms post word onset and shows a larger amplitude for two vs. one referent situations. Clearly the negativity elicited in the present experiment has a different scalp distribution and does not show any frontal focus. We therefore categorized our negativity as an N400.

^5^By strengthening certain parts of the active semantic network.
