# Supplementary material for: Inconsistent use of gesture space during abstract pointing impairs language comprehension
Source: Front Psychol. 2015 Feb 9;6:80. doi: 10.3389/fpsyg.2015.00080 (PMC4321330; doi:10.3389/fpsyg.2015.00080)
Supplement: Supplementary file 1 [file Presentation1.ZIP › ExampleOfConsistentPointingInGestureSpace_3.pptx]

## Slide 1
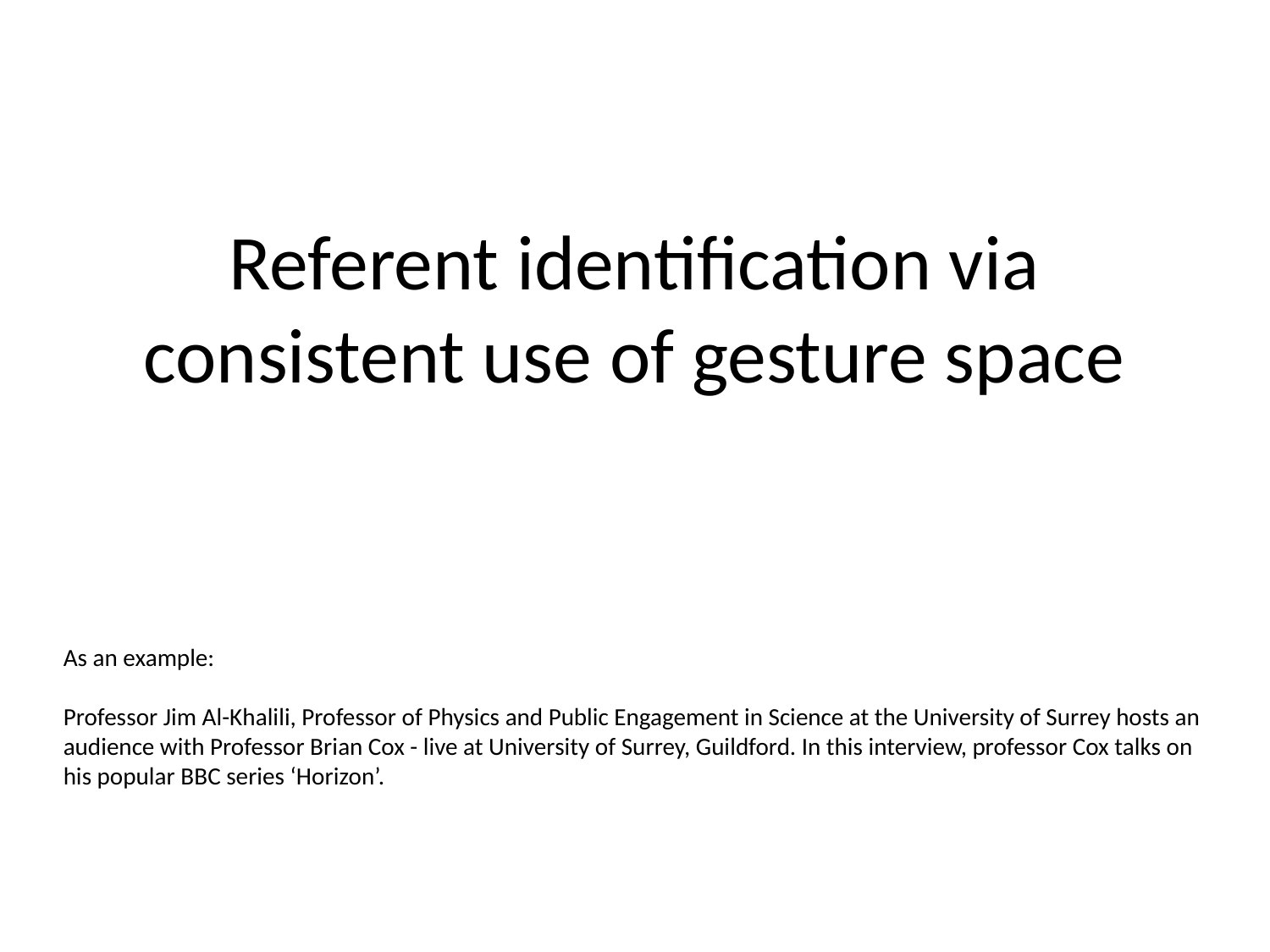

Referent identification via consistent use of gesture space
As an example:
Professor Jim Al-Khalili, Professor of Physics and Public Engagement in Science at the University of Surrey hosts an audience with Professor Brian Cox - live at University of Surrey, Guildford. In this interview, professor Cox talks on his popular BBC series ‘Horizon’.

## Slide 2
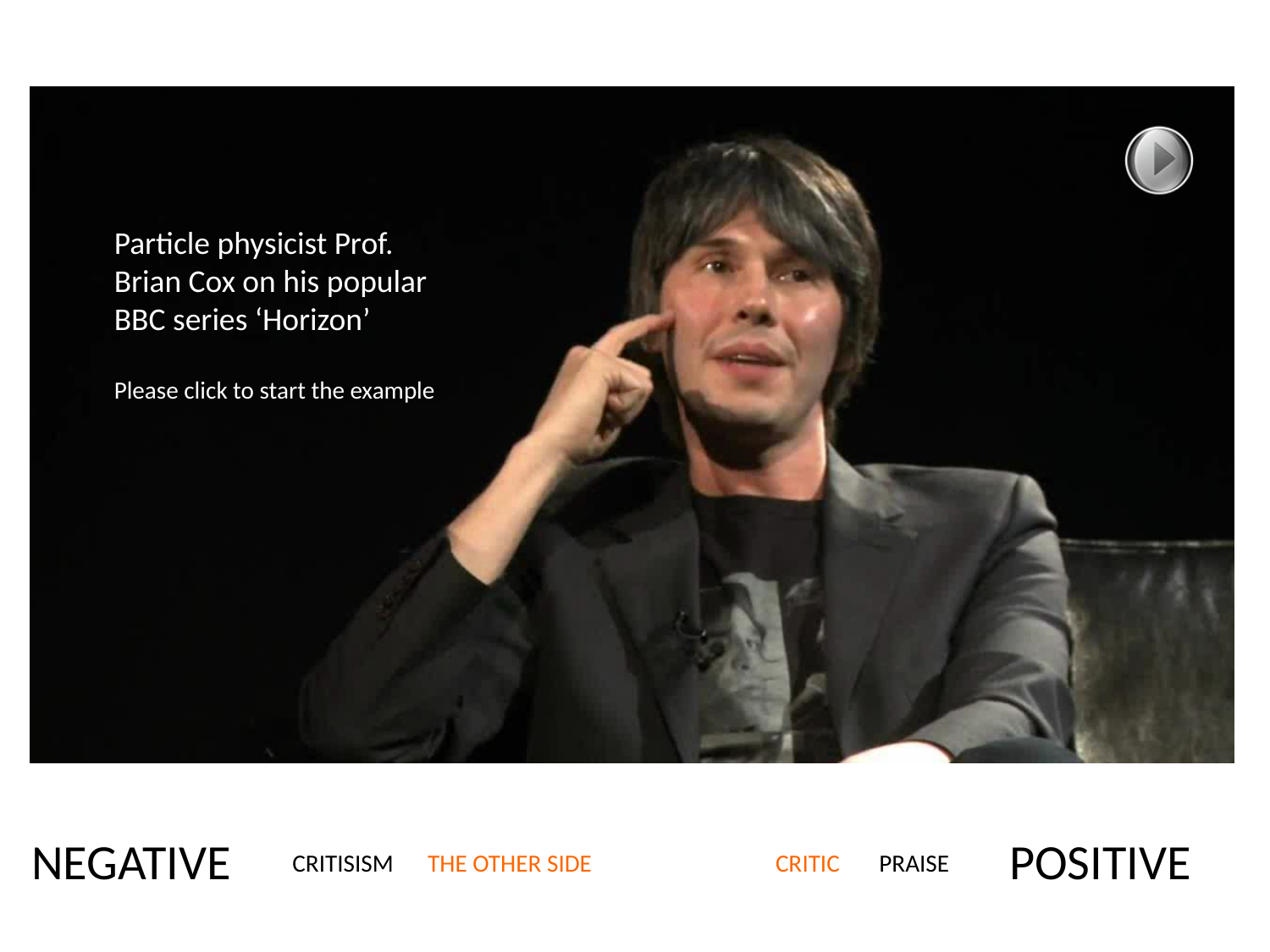

Particle physicist Prof. Brian Cox on his popular BBC series ‘Horizon’
Please click to start the example
NEGATIVE
POSITIVE
CRITISISM
THE OTHER SIDE
CRITIC
PRAISE

## Slide 3
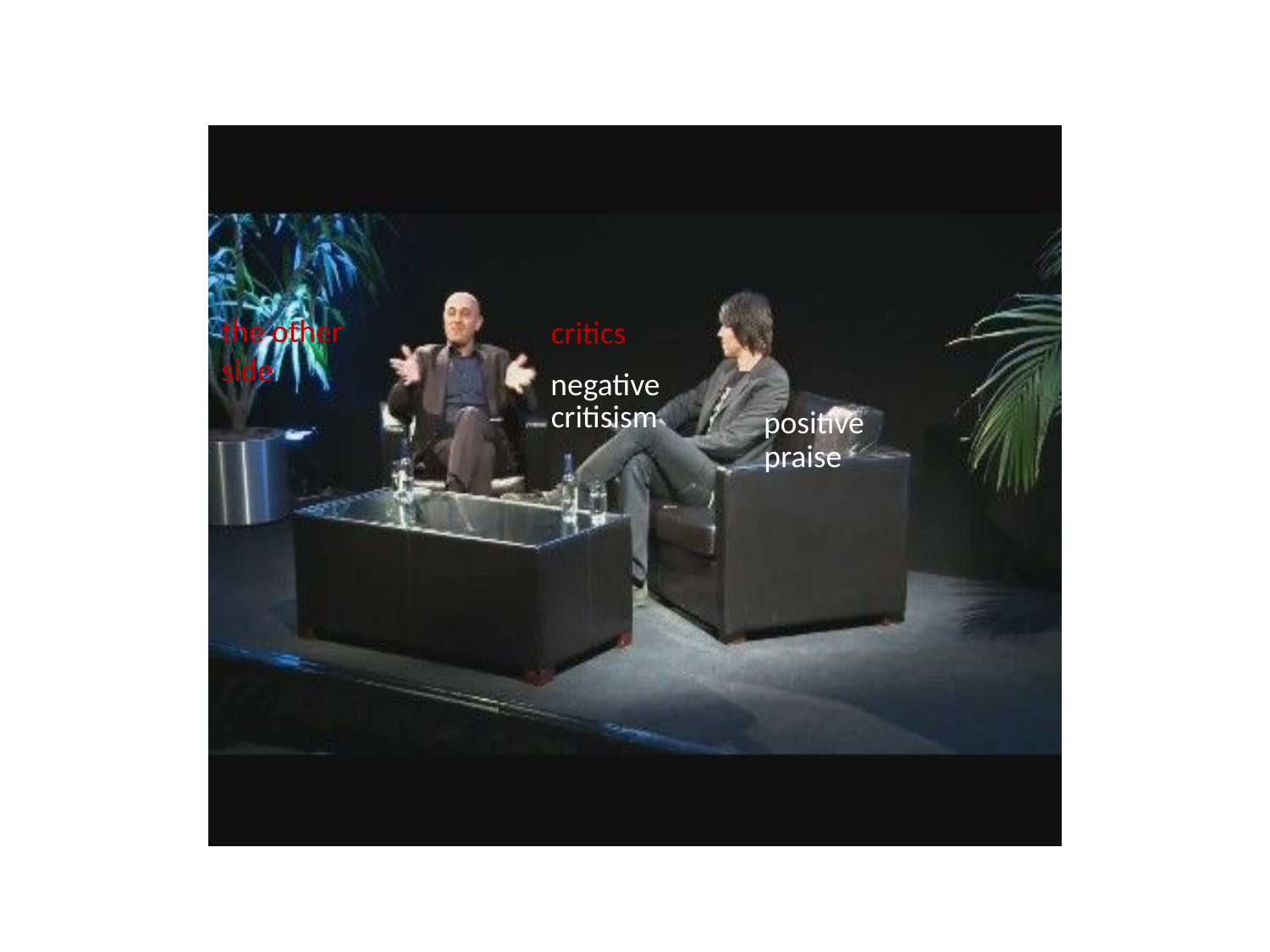

the other side
critics
negative
critisism
positive
praise

## Slide 4
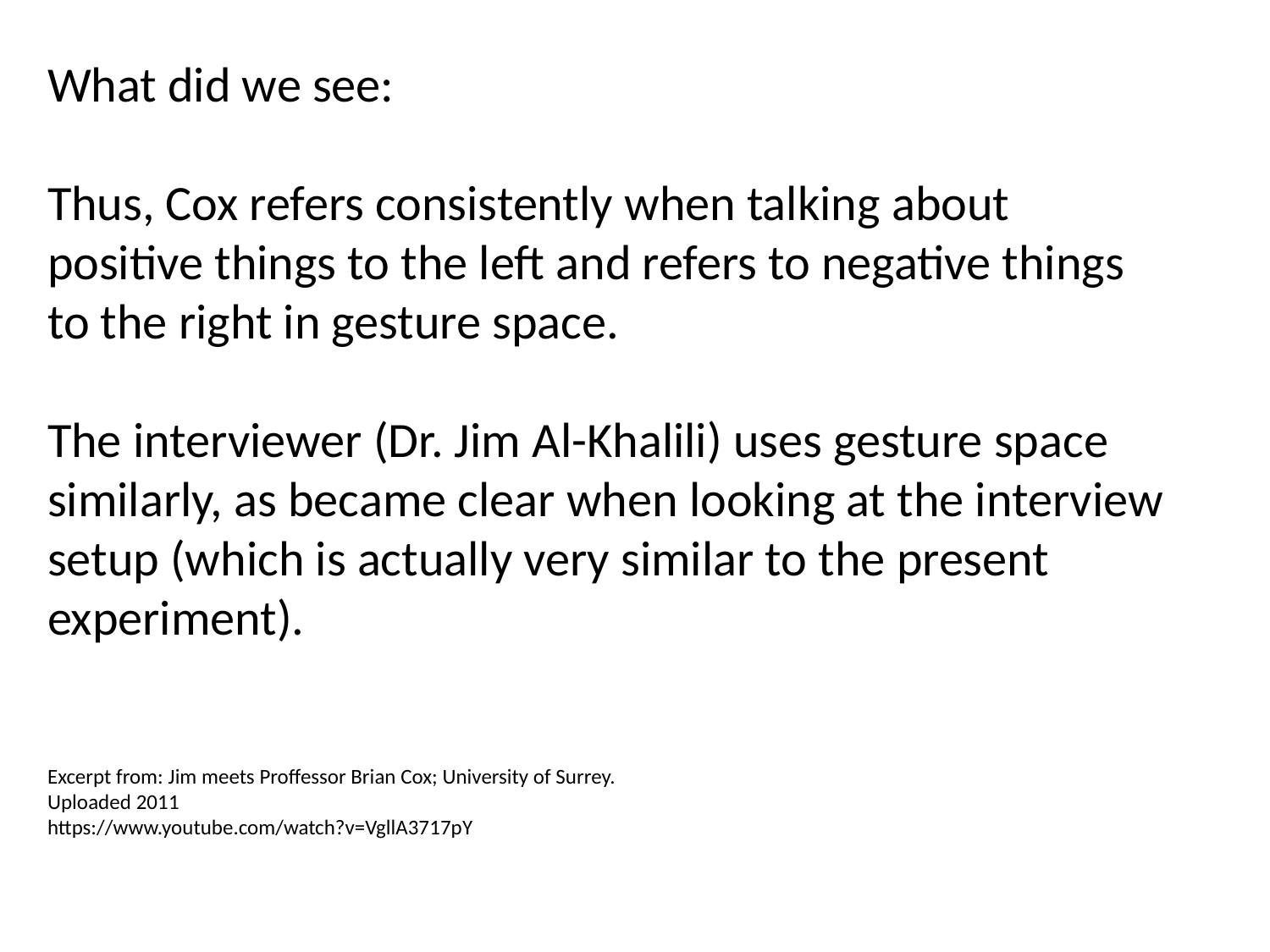

What did we see:
Thus, Cox refers consistently when talking about positive things to the left and refers to negative things to the right in gesture space.
The interviewer (Dr. Jim Al-Khalili) uses gesture space similarly, as became clear when looking at the interview setup (which is actually very similar to the present experiment).
Excerpt from: Jim meets Proffessor Brian Cox; University of Surrey.
Uploaded 2011
https://www.youtube.com/watch?v=VgllA3717pY
