# Supplementary figures and images for: Inconsistent use of gesture space during abstract pointing impairs language comprehension
Source: Front Psychol. 2015 Feb 9;6:80. doi: 10.3389/fpsyg.2015.00080 (PMC4321330; doi:10.3389/fpsyg.2015.00080)

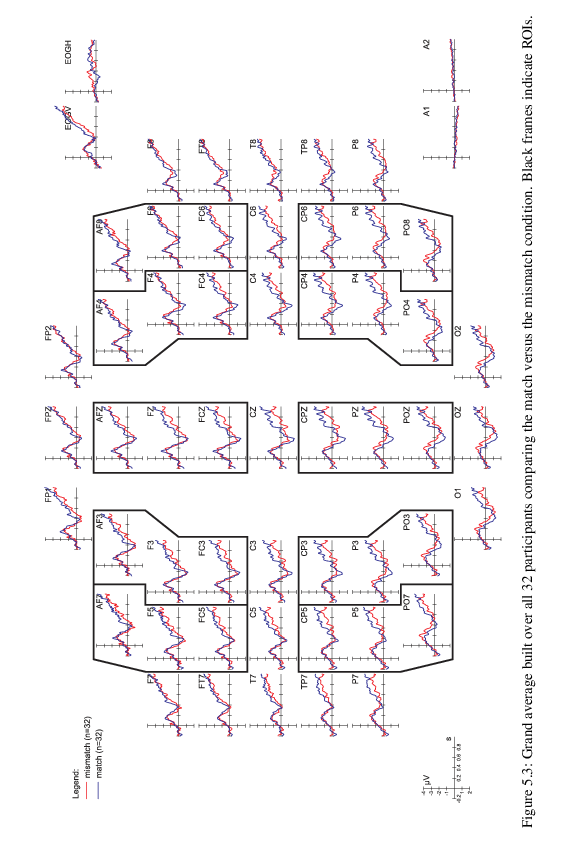

Supplement: Supplementary file 2 [file Image1.PNG]
